# A Novel Secreted Protein-Related Gene Signature Predicts Overall Survival and Is Associated With Tumor Immunity in Patients With Lung Adenocarcinoma

**DOI:** 10.3389/fonc.2022.870328

**Published:** 2022-06-03

**Authors:** Shuaijun Chen, Jun Zhang, Qian Li, Lingyan Xiao, Xiao Feng, Qian Niu, Liqin Zhao, Wanli Ma, Hong Ye

**Affiliations:** ^1^ Department of Pathophysiology, School of Basic Medicine, Tongji Medical College, Huazhong University of Science and Technology, Wuhan, China; ^2^ Department of Obstetrics and Gynecology, Union Hospital, Tongji Medical College, Huazhong University of Science and Technology, Wuhan, China; ^3^ Department of Oncology, Tongji Hospital, Tongji Medical College, Huazhong University of Science and Technology, Wuhan, China; ^4^ Department of Respiratory and Critical Care Medicine, Union Hospital, Tongji Medical College, Huazhong University of Science and Technology, Wuhan, China; ^5^ Key Laboratory of Respiratory Diseases, National Health Commission of China, Wuhan, China

**Keywords:** lung adenocarcinoma, secretome, secreted protein-related risk score, gene signature, immune landscape

## Abstract

Secreted proteins are important proteins in the human proteome, accounting for approximately one-tenth of the proteome. However, the prognostic value of secreted protein-related genes has not been comprehensively explored in lung adenocarcinoma (LUAD). In this study, we screened 379 differentially expressed secretory protein genes (DESPRGs) by analyzing the expression profile in patients with LUAD from The Cancer Genome Atlas database. Following univariate Cox regression and least absolute shrinkage and selection operator method regression analysis, 9 prognostic SPRGs were selected to develop secreted protein-related risk score (SPRrisk), including CLEC3B, C1QTNF6, TCN1, F2, FETUB, IGFBP1, ANGPTL4, IFNE, and CCL20. The prediction accuracy of the prognostic models was determined by Kaplan–Meier survival curve analysis and receiver operating characteristic curve analysis. Moreover, a nomogram with improved accuracy for predicting overall survival was established based on independent prognostic factors (SPRrisk and clinical stage). The DESPRGs were validated by quantitative real-time PCR and enzyme-linked immunosorbent assay by using our clinical samples and datasets. Our results demonstrated that SPRrisk can accurately predict the prognosis of patients with LUAD. Patients with a higher risk had lower immune, stromal, and ESTIMATE scores and higher tumor purity. A higher SPRrisk was also negatively associated with the abundance of CD8^+^ T cells and M1 macrophages. In addition, several genes of the human leukocyte antigen family and immune checkpoints were expressed in low levels in the high-SPRrisk group. Our results provided some insights into assessing individual prognosis and choosing personalized treatment modalities.

## Introduction

Lung cancer is the main cause of cancer-related deaths worldwide and accounts for approximately one-quarter of all cancer-related deaths, 82% of which are directly caused by cigarette smoking (1). Lung adenocarcinoma (LUAD) is the most common histological type, accounting for nearly 40% of all lung cancer cases (2). Despite intensive research and the development of several new targeted agents and immunotherapies, the survival rates for patients with LUAD remain dismal. The 5-year survival rate of lung adenocarcinoma is only 4–17% (3). Over 60% of patients with lung cancer are not diagnosed until the late stages of the disease (4). Therefore, early detection and personalized treatment may significantly improve patient survival.

Nowadays, with the rapid development of high-throughput sequencing technologies, many bioinformatics studies aim to identify biomarkers that can establish prognosis or predict drug response in patients with cancer (5, 6). Despite these advances, several critical limitations remain to be addressed. First, it is difficult to obtain tumor tissue samples. Second, performing transcriptome sequencing is expensive. These issues limit the use of combined gene signature models on a larger scale. Moreover, the available tumor tissues are usually from the intermediate or advanced stages of tumor progression, indicating missed opportunities for early detection and clinical intervention. Therefore, it is necessary to find an easy and attractive method to evaluate the prognosis of patients with LUAD.

In recent years, a large-scale, high-throughput protein expression, purification, and screening platform has been developed, establishing a secreted protein library (7). Over 2,000 human genes have been reported to encode known secreted proteins, including hormones, cytokines, proteases, antibodies, poison, and growth factors (8, 9). The proteins were classified into three major categories: (i) blood proteins, (ii) locally secreted proteins, and (iii) intracellular proteins (10). These proteins play important physiological roles in various biological processes, such as cell signal transduction, adhesion, migration, and immune defense (11–13). Meanwhile, several secreted proteins in diseases might serve as early-stage diagnostic and prognostic markers. These secreted proteins are also considered as new therapeutic agents or as targets for small molecule or antibody drug development (14)—for example, anterior gradient-2 (AGR2) is a secreted protein reported to be highly expressed in a variety of tumor types. Thus, AGR2 is related to the proliferation, metastasis, invasion, and drug resistance of tumor cells, making it an attractive target for early diagnosis and tumor therapy (15–17). Additionally, IL-6 plays a critical role in chronic inflammation, autoimmune diseases, infectious diseases, metabolic diseases, and cancer, and thus the IL-6 cytokine family has been used as a diagnostic or prognostic indicator of disease activity and response to therapy (18–21). Moreover, the IL-6 family of cytokines is now regarded as a major therapeutic target for clinical interventions (18, 20, 22, 23). Therefore, delving into the study of secreted proteins allows clinicians to evaluate the prognosis of patients with early-stage diseases and holds promise for individualized therapeutic interventions.

With regard to LUAD, a series of secreted proteins have been reported to be dysregulated and involved in LUAD progression. Widely used serum tumor markers, such as carcinoembryonic antigen, carbohydrate antigen 199, and neuron-specific enolase, have been used for the early diagnosis and classification of lung cancer (24). Many chemokines have been implicated in the modulation of the immune response, which has diverse functions in LUAD. It has been reported that CXCL17 expression in lung cancer cells could promote tumor progression (25). In addition, CCL20 was upregulated in patients with relapsed lung cancer and could accelerate cell proliferation through the ERK signaling pathway (26). Higher serum levels of IL-22 and HGF were observed in patients with non-small cell lung cancer (NSCLC) than in healthy subjects. Elevated serum IL-22 and loss of IL-34 expression have been associated with a poor prognosis in patients with NSCLC and LUAD, respectively (27). Pang *et al*. reported that RCC2 overexpression could induce JNK activation and upregulate MMPs (such as MMP-1, MMP-2, and MMP-9), which belong to a family of metastasis-related secretory proteins, in LUAD (28). To the best of our knowledge, a systematic investigation of secreted proteins in LUAD has not been reported.

Since the detection of secreted proteins is convenient, economical, and a minimally invasive intervention, developing a prognostic signature of secreted protein-related genes (SPRGs) is of great interest. In this study, we aimed to develop a useful tool to evaluate the prognostic role of secreted protein-related risk score (SPRrisk) based on large-scale RNA-seq data for LUAD from The Cancer Genome Atlas (TCGA) cohort and Gene Expression Omnibus (GEO) databases. We further used least absolute shrinkage and selection operator method (LASSO) regression and multivariate Cox regression analyses to investigate potential secreted protein-related prognostic genes and constructed SPRrisk to predict survival in patients with LUAD.

## Materials and Methods

### Data Acquisition

The LUAD level 3 RNA-seq data (read counts) and corresponding clinical information of 535 tumor samples and 59 normal samples were downloaded from TCGA (https://portal.gdc.cancer.gov/) as a training cohort, and the ENSEMBL gene ID was converted into a gene name for the subsequent analysis. The LUAD microarray data GSE72094 (*n* = 442) and GSE31210 (*n* = 246) were downloaded with complete clinical data from GEO (http://www.ncbi.nlm.nih.gov/geo) to serve as the validation sets. GSE72094 was from the chip platform GPL15048 (Rosetta/Merck Human RSTA Custom Affymetrix 2.0 microarray) (29), and the CEL files were normalized against their median sample using the IRON algorithm (30). GSE31210 was from the chip platform GPL570 (Affymetrix Human Genome U133 Plus 2.0 Array) (31), and the mRNA expression data were normalized by the MAS5 algorithm (32). All the genes detected with more than one probe were calculated by mean expression, and the gene expression data were log2-transformed before the analyses. Despite the large number of secreted proteins, only those secreted into the plasma were selected for our study. Finally, a total of 730 secreted protein-related genes (SPRGs) ultimately remained. The SPRG list was retrieved from the HPA database (https://www.proteinatlas.org/humanproteome/blood+protein/secreted+to+blood) and is provided in **Supplementary Table S1**.

### Construction and Validation of a Prognostic Secreted Protein-Related Gene Signature

Using the RNA-Seq data of TCGA LUAD dataset and the list of SPRGs obtained as detailed above, we finally got the SPRGs expression profiles of patients with LUAD. TCGA LUAD read count data of SPRGs were then processed with the “edgeR” R package (version 3.36.0) for normalization and differential expression analysis. The following criteria were applied to filter differentially expressed secreted protein-related genes (DESPRGs) between tumor tissues and normal tissues: false discovery rate (FDR) <0.05 and |log2 fold change| >1 (33). Following this, univariate Cox regression analysis was applied to identify SPRGs with prognostic values using the “survival” package of the R software. Next, we conducted LASSO regression to narrow the range of prognostic genes, removed overfitting between genes, and calculated risk scores according to LASSO regression coefficients with the “glmnet” R package (34, 35). Therefore, a final model with 9 variables was obtained at the end. The risk score of each patient was calculated by multiplying the gene expression by the regression coefficient. The formula was established as follows: Secreted protein-related signature (SPRS) = Σ*_i_β_i_* * *Exp_i_*. The final risk model was as follows: SPRGrisk = (-0.0290 ∗ CLEC3B expression) + (0.1830 ∗ C1QTNF6 expression) + (0.0020 ∗ TCN1 expression) + (0.0123 ∗ F2 expression) + (0.0522 ∗ FETUB expression) + (0.0381 ∗ IGFBP1 expression) + (0.0185 ∗ ANGPTL4 expression + (0.0107 ∗ IFNE expression) + (0.0051 ∗ CCL20 expression). The expression level of each gene was log2-normalized.

### Evaluation of the Predictive Efficacy of the Prognostic Model

All patients were classified as high or low risk based on their median risk score, and survival curves were used to assess the predictive power of the prognostic model between the high- and low-risk groups with “survminer” R package. “timeROC” package was applied to evaluate the prognostic model’s ability to predict outcomes in patients with LUAD (36), and the areas under the curve (AUC) at different time points of all the variables were compared. Principal component analysis (PCA) and t-distributed stochastic neighbor embedding (t-SNE) were performed to explore the distribution of the high- and low-risk groups using the “Rtsne” R package, with the expression matrix of the 9 selected genes as the input. Given the presence of correlation between SPRrisk and other risk factors (stage, gender, and TP53 mutation), to make the results more convincing, we performed the additional multivariate Cox analyses after adjustments for other clinicopathologic characteristics. The detailed method was as follows: after removing the clinicopathologic parameter that needs to be adjusted, multivariate Cox analysis was performed on the remaining parameters, including SPRrisk. To evaluate the proposed SPRG model in comparison with other models, different risk scores were calculated for each patient using different models, and the AUC at different time points of all the different risk scores was drawn on the same set of coordinate axes for comparison.

### Construction and Evaluation of a Nomogram

To identify the best prognostic indicators of the survival outcome of LUAD patients, univariate and multivariate Cox regression analyses were performed. Finally, variables whose *p*-value was less than 0.05 (*p* < 0.05) were selected to build a nomogram. The nomogram was constructed and evaluated by employing the R packages “rms”, “regplot”, and “Hmisc”. Moreover, the concordance index (C-index) and calibration curve were adapted to appraise the availability of this nomogram in both the training set and the validation set. The ROC analysis was also performed to assess the accuracy of the nomogram for 1-, 3-, and 5-year overall survival of patients with LUAD.

### Characterization of the Immune Cell Landscape and the Prediction of Therapeutic Sensitivity in Patients With LUAD

The ESTIMATE algorithm was adopted to estimate the immune scores and stromal scores of LUAD patients with the R package “estimate” (37). In order to determine the composition of immune cells in the tumor microenvironment of each sample, we performed deconvolution with support vector regression using the CIBERSORT algorithm. The LM22 immune cell signature matrix was downloaded from the CIBERSORT website (https://cibersort.stanford.edu/). CIBERSORT was run for 1,000 permutations, and quantile normalization was applied. The potential response of patients with LUAD to immunotherapy was evaluated by the tumor immune dysfunction and exclusion (TIDE) score and immunophenoscore (IPS). Data is available for download from TIDE (http://tide.dfci.harvard.edu/) or The Cancer Immunome Atlas (TCIA) (https://tcia.at/home) (38, 39).

### Tissue Samples and Quantitative Real-Time Polymerase Chain Reaction

All tissue and blood samples were collected from the Thoracic Surgery Department of Wuhan Union Hospital, which was approved by the Medical Ethics Committee of the hospital. Written informed consent was obtained from each involved patient. A total of 25 lung adenocarcinoma tissue samples and 25 non-tumor lung tissues were obtained from the tumor and adjacent tissue of lung adenocarcinoma patients who underwent tumor resection between October 2019 and July 2021. All included patients were newly diagnosed and had not received any relevant treatment prior to surgery, and follow-up started at the date of diagnosis and ended at death or on October 31, 2021. For gene expression studies, the total RNA from tissues was isolated with TRIZOL reagents (Takara, Otsu, Japan). RNA extraction was performed according to the manufacturer’s protocols. RNA was reverse-transcribed into cDNA by RT-PCR using Hiscript@ Q RT SuperMix (Vazyme, Nanjing, China) in a 20-μl total sample volume. The parameters of reaction were as follows: 95°C for 30 s, followed by 40 cycles at 95°C for 5 s and 60°C for 1 min. Then, the gene expression levels were measured by quantitative PCR (qPCR). qPCR was performed in a CFX Connect Real-Time PCR Detection System (Bio-Rad, Hercules, CA, USA) using SYBR green supermix (Vazyme, Nanjing, China). The total amount of mRNA was normalized to endogenous GAPDH mRNA. The 2^-ΔΔCt^ method was used to calculate the related gene expression levels. The primer sequences are listed in **Supplementary Table S2**.

### Enzyme-Linked Immunosorbent Assay

We used enzyme-linked immunosorbent assay (ELISA) kits to measure the plasma levels of FETUB [Human Fetuin B (FETUB) ELISA Kit; Reddot Biotech], IGFBP1 [Human Insulin Like Growth Factor Binding Protein 1 (IGFBP1) ELISA Kit; Reddot Biotech], TCN1 [Human Transcobalamin I (TCN1) ELISA Kit; Reddot Biotech], ANGPTL4 [Human Angiopoietin Like Protein 4 (ANGPTL4) ELISA Kit; Reddot Biotech], and CCL20 (Human CCL20/MIP-3 alpha ELISA Kit, Proteintech). Approximately 1 ml of blood was collected in EDTA-coated tubes on ice (BD Vacutainer), centrifuged at 4°C (2,000 × *g*, 10 min), aliquoted, and stored at −20°C until the assay was performed using the ELISA kits. Subsequent steps were carried out following the manufacturer’s protocol.

### Statistical Analysis

All statistical analyses were conducted using R version 4.0.0 (2020-04-24). To test for differential expression across two groups (tumor and normal), the *p*-values were adjusted for multiple testing based on the FDR according to the Benjamini–Hochberg approach. The survival analysis was performed using the Kaplan–Meier (KM) method, and the subgrouping of the samples was stratified by medians of gene expression levels. Student’s *t*-test or one-way analysis of variance was used to analyze differences between groups in variables with a normal distribution. Differences in proportions were compared by chi-square test. If not specified above, a *P*-value less than 0.05 was considered statistically significant, and all *P*-values were two-tailed.

## Results

### Identification of Differentially Expressed Secreted Protein-Related Genes in the Cancer Genome Atlas Training Cohort

**Figure 1:**
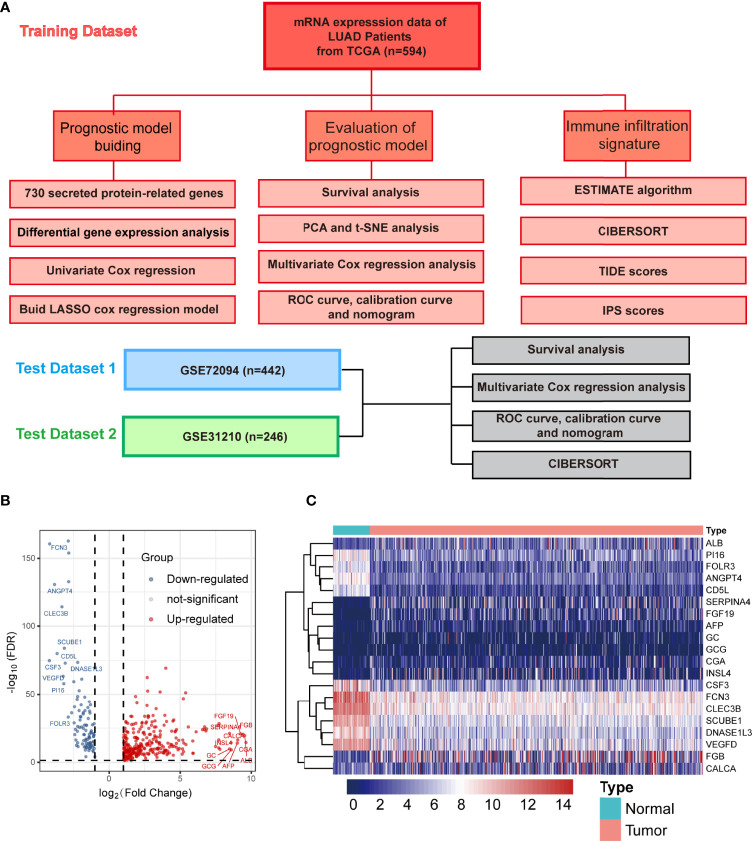
Screening for secreted protein-related genes in The Cancer Genome Atlas lung adenocarcinoma cohort. **(A)** Flow diagram of the research process. **(B)** Volcano plot of differentially expressed secretory protein genes (DESPRGs) between lung adenocarcinoma and normal lung tissues; the top 10 genes with the highest and lowest expression are labeled respectively. **(C)** Heat map diagram of the top 10 DESPRGs in lung adenocarcinoma compared with normal lung tissue. Red indicates a high expression, while blue indicates a low expression.

A flow chart was developed to systematically describe our study (**Figure 1A**). We firstly obtained the gene expression profiles of the SPRGs from TCGA LUAD dataset. The gene list was compiled from the literature and the HPA database, which included 730 genes encoding the proteins secreted into plasma. To screen for DESPRGs, differential expression analysis of the data was performed using the edgeR software. Finally, we obtained 379 DESPRGs, including 281 upregulated and 98 downregulated SPRGs (**Figure 1B**). The criteria to indicate a significant differential expression were as follows: |log2-fold change| >1 and FDR <0.05. The most obvious genes with an elevated expression were *CGA*, *ALB*, *FGB*, *FGF19*, *CALCA*, alpha fetoprotein (*AFP*), *GCG*, *INSL4*, *GC*, and *SERPINA4*. The most significantly downregulated genes included *CSF3*, *FCN3*, *ANGPT4*, *CD5L*, *CLEC3B*, *VEGFD*, *PI16*, *SCUBE1*, *DNASE1L3*, and *FOLR3* (**Figure 1C**). Evidently, a large number of SPRGs encoding proteins secreted into the plasma were differentially expressed in LUAD compared with those in adjacent normal samples. Among these genes, *AFP* and *VEGFD* are well-known tumor biomarkers that have been extensively applied in the early screening of tumors (40, 41). *FGF19* has been implicated in the pathogenesis of several cancers, including hepatocellular carcinoma in mice and potentially in humans (42). Some of these DESPRGs belong to the endocrine signaling pathway (including *CGA* and *GCG*), whereas some DESPRGs are involved in the regulation of the immune system process (including *CSF3* and *CD5L*). These results altogether suggested that these SPRGs had a potential prognostic value in patients with LUAD.

### Establishment of Secreted Protein-Related Gene Signatures for Prognosis

**Figure 2:**
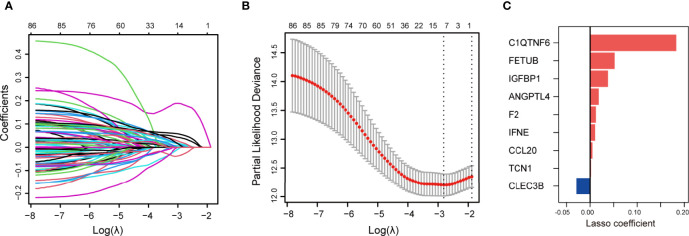
Construction of secreted protein-related gene (SPRG)-related model for patients with lung adenocarcinoma (LUAD). **(A)** Least absolute shrinkage and selection operator method (LASSO) coefficient spectrum of differentially expressed secretory protein genes in The Cancer Genome Atlas LUAD cohort. **(B)** Cross-validation fit curve calculated by LASSO regression method. **(C)** Distribution of LASSO coefficients of the selected SPRGs.

Next, the prognostic role of SPRGs in patients with LUAD was examined. Using univariate Cox regression analysis on the stated DESPRGs, we identified 86 overall survival-associated genes in the samples of patients with LUAD in TCGA cohort (*P* < 0.05) (**Supplementary Figure S1**). Subsequently, we performed LASSO Cox regression analysis to identify the most robust marker genes for prognosis. Tenfold cross-validation was applied to prevent over-fitting, with a selected optimal *λ* value of 0.0602 (**Figures 2A, B**). Finally, an ensemble of 9 genes (*CLEC3B*, *C1QTNF6*, *TCN1*, *F2*, *FETUB*, *IGFBP1*, *ANGPTL4*, *IFNE*, and *CCL20*) was identified. The genes’ individual nonzero LASSO coefficients and the distribution of LASSO coefficients of the gene signature are shown in **Figure 2C**. Meanwhile, KM survival analysis was performed for each gene separately, and the survival curves were plotted. The results indicated that patients with LUAD with a high expression of *C1QTNF6*, *TCN1*, *F2*, *FETUB*, *IGFBP1*, *ANGPTL4*, *IFNE*, and *CCL20* had a poor prognosis, whereas patients with a high expression of *CLEC3B* had a better prognosis (**Supplementary Figures S2A–I**). The patients’ risk scores were calculated from the expression levels and regression coefficients: SPRrisk = (-0.0290 ∗ CLEC3B expression) + (0.1830 ∗ C1QTNF6 expression) + (0.0020 ∗ TCN1 expression) + (0.0123 ∗ F2 expression) + (0.0522 ∗ FETUB expression) + (0.0381 ∗ IGFBP1 expression) + (0.0185 ∗ ANGPTL4 expression + (0.0107 ∗ IFNE expression) + (0.0051 ∗ CCL20 expression). The expression level of each gene was log2-normalized.

### SPRrisk Acts as an Indicator of Unfavorable Outcome in TCGA Training Cohort

**Figure 3:**
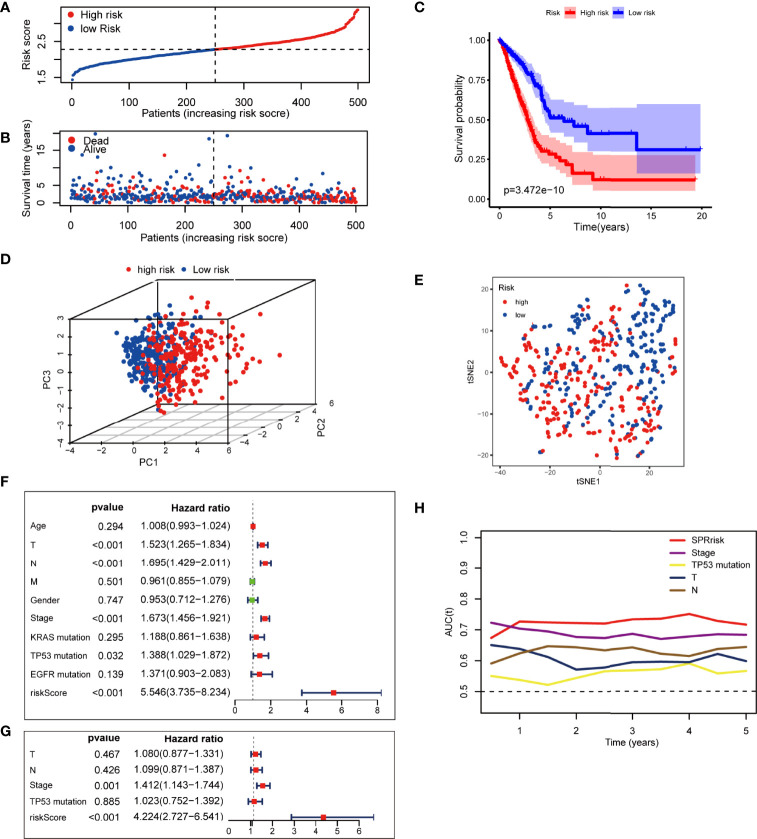
Assessment of the prognostic signature in The Cancer Genome Atlas (TCGA) testing cohort. **(A)** Distribution of risk score and survival time of patients with lung adenocarcinoma (LUAD). **(B)** Scatter plot of survival status and risk score in patients with LUAD. **(C)** Kaplan–Meier curves of overall survival time between high- and low-risk groups using the log-rank test in TCGA LUAD dataset. **(D)** Principal component analysis of the 9 SPRG expression profiles of the high- and low-risk groups. **(E)** t-SNE analysis of the 9 SPRG expression profiles of the high- and low-risk groups as indicated by different colors. **(F, G)** Forest plot with hazard ratios from the univariate and multivariable Cox proportional hazards regression analysis in TCGA cohort. **(H)** The areas under the curve of time-dependent receiver operating characteristic curves verified the prognostic performance of the risk score in TCGA cohort.

To further investigate the prognosis value of SPRrisk, we classified the patients with LUAD in TCGA training cohort into high-risk and low-risk groups based on the median SPRrisk score (**Figure 3A**). The scatter plot of risk scores and survival status indicated that poor prognostic outcomes were more common in the high-risk group, whereas the low-risk group had a significantly longer survival time (**Figure 3B**). The KM survival curves revealed that the prognosis of patients with low SPRrisk was significantly better in TCGA training cohort (*P* < 0.01) (**Figure 3C**). Furthermore, the prognostic value of SPRS in predicting disease-free interval (DFI), disease-specific survival (DSS), and progression-free interval (PFI) was reasonably consistent; the low-risk patients tended to have better DFI, DSS, and PFI values (**Supplementary Figures S3A–C**) than the high-risk patients. Moreover, PCA and t-SNE analyses showed that the patients were distributed in two subgroups in a discrete direction based on nine DESPRGs recognized by the LASSO Cox regression analysis (**Figures 3D, E**).

To assess whether risk score was an independent prognostic factor for LUAD, we performed univariate and multivariate Cox regression analyses for the SPRrisk and other risk factor variables (age, TNM classification, gender, stage, KRAS mutation, TP53 mutation, and EGFR mutation) in TCGA training cohort. The univariate Cox regression results indicated that the patients’ risk scores were significantly associated with overall survival (OS) (HR = 5.546, 95%CI = 3.735–8.234, *P* < 0.001) (**Figure 3F**). In the multivariate Cox regression analysis, SPRrisk was proved to be an independent risk factor for OS in TCGA training cohort (HR = 4.224, 95%CI = 2.727–6.541, *P* < 0.001) (**Figure 3G**). Furthermore, the results of the time–ROC analysis showed that SPRrisk was the most accurate predictor for OS (**Figure 3H**).

### SPRrisk Is Closely Related to Different Clinicopathological Features

**Figure 4:**
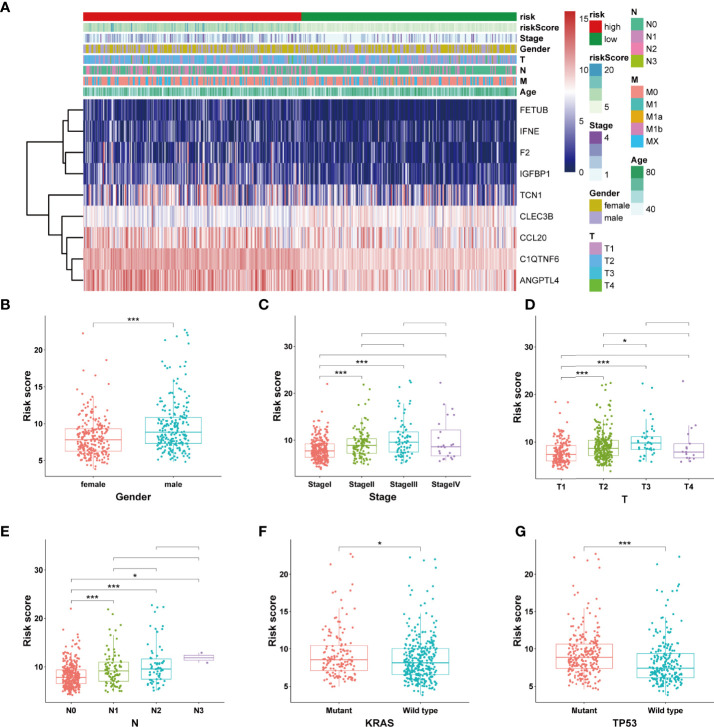
Estimation of the correlation between the SPRrisk and different clinicopathological features. **(A)** Heat map showing the association of the expression levels of 9 selected secreted protein-related genes and clinicopathologic features. **(B–G)** The different levels of risk scores in lung adenocarcinoma patients were stratified by gender, clinical stage, T stages, N stages, KRAS mutation status, and TP53 mutation status. *P < 0.05, ***P < 0.001.

The expression levels of the nine SPRGs in the high- and low-risk groups in TCGA cohort LUAD dataset are presented in a heat map (**Figure 4A**). We also analyzed the association between the patients’ risk scores and other pathological features in TCGA LUAD dataset. There were significant differences in the risk scores between patients of different gender (*P* < 0.05), clinical stage (*P* < 0.01), T stage (*P* < 0.01), KRAS mutation status (*P* < 0.05), and TP53 mutation status (*P* < 0.01) (**Figures 4B–G** and **Table 1**). The correlations among the SPRrisk, clinical stage, T stage, and TP53 mutation status partly revealed why SPRrisk could be a better prognostic marker in predicting OS for LUAD patients. Moreover, the incidence of LUAD is higher in women, and lung cancer in women is a severe health problem globally (43). LUAD is considered a different disease in women and men (43). However, the effect of sex on LUAD patients’ survival is still controversial. In our study, men had a higher SPRrisk than women in TCGA cohort; this result may be due to differences in secretion environments between males and females or due to a greater exposure to risk factors in men. A more conclusive explanation requires further studies.

**Table 1:** Clinical and pathological characteristics of high- and low-risk patients in The Cancer Genome Atlas lung adenocarcinoma data set.

Parameter	SPRrisk-high	SPRrisk-low	*P*-value
	(*N* = 250)	(*N* = 250)	
Age (years)			
Mean (SD)	63.8 (10.4)	66.7 (10.4)	0.0015
Median (min., max.)	64 (33.0, 87.0)	68.0 (41.0, 88.0)	
Pathologic T			
T1	59 (23.6%)	108 (43.2%)	<0.001
T2	150 (60.0%)	117 (46.8%)	
T3	32 (12.8%)	13 (5.2%)	
T4	8 (3.2%)	10 (4.0%)	
Pathologic N			
N0	137 (54.8%)	187 (74.8%)	<0.001
N1	60 (24.0%)	34 (13.6%)	
N2	48 (19.2%)	21 (8.4%)	
N3	2 (0.8%)	0 (0%)	
Pathologic M			
M0	164 (65.6%)	168 (67.2%)	0.699
M1	14 (5.6%)	10 (4.0%)	
MX	70 (28.0%)	70 (28.0%)	
Gender			
Male	118 (47.2%)	112 (44.8%)	0.654
Female	132 (52.8%)	138 (55.2%)	
Stage			
Stage I	103 (41.2%)	165 (66.0%)	<0.001
Stage II	76 (30.4%)	43 (17.2%)	
Stage III	53 (21.2%)	27 (10.8%)	
Stage IV	14 (5.6%)	11 (4.4%)	
KRAS mutation			
WT	163 (65.2%)	175 (70.0%)	0.281
MUT	81 (32.4%)	69 (27.6%)	
TP53 mutation			
WT	88 (35.2%)	145 (58.0%)	<0.001
MUT	156 (62.4%)	99 (39.6%)	
EGFR mutation			
WT	216 (86.4%)	210 (84.0%)	0.497
MUT	28 (11.2%)	34 (13.6%)	

### High SPRrisk Reflects the Low Level of Immune Infiltration in LUAD

**Figure 5:**
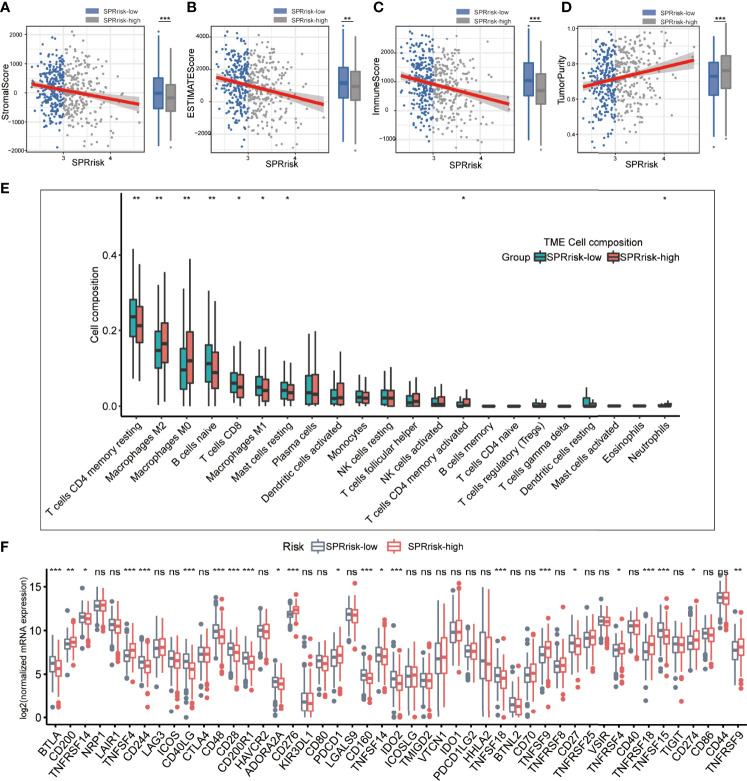
Landscape of immune and stromal cell infiltrations in the low- and high-risk groups. **(A–D)** Comparison of ESTIMATE scores, immune scores, stromal scores, and tumor purity between the high- and low-risk groups. **(E)** The immune cell infiltration levels of 22 immune cell types between the low- and high-risk groups for patients with lung adenocarcinoma. **(F)** Analyses for the expression of immune checkpoint genes in the high- and low-risk groups. *P < 0.05, **P < 0.01, ***P < 0.001, and ns (no significance).

Since many cytokines are secreted proteins and participate in the regulation of the tumor microenvironment, we analyzed the differences in their composition between the high- and low-risk groups. Firstly, using the ESTIMATE algorithm, we observed that the low-risk group had higher ESTIMATE, immune, and stromal scores and lower tumor purity than the high-risk group, suggesting that the tumor cells in the low-risk group had more immune cell infiltration (**Figures 5A–D**). Moreover, we used CIBERSORT on RNA-seq gene expression profiles to quantify the relative abundance of 22 different immune cell types in the tumor immune microenvironment. The results revealed that the SPRrisk-low group had high levels of multiple antitumor immune components, including M1 macrophages and CD8^+^ T cells, while the proportion of M2 macrophages was higher in the SPRrisk-high group (**Figure 5E**). We also examined the expression of immunomodulatory genes between the high- and low-risk groups and found that high-risk patients had higher levels of pro-tumorigenic immunomodulatory molecules (including CD274 and CD276) and lower levels of anti-tumorigenic immunomodulatory molecules (including CD40LG and TNFRSF14) (**Figure 5F**). Human leucocyte antigen (HLA) complexes control the adaptive immunity by delivering defined fractions of intracellular and extracellular protein content to immune cells and have been shown to play important roles in anti-tumor immunity (44). In the present study, we also observed lower levels of HLA complexes in high-risk patients, including *HLA-DPB2*, *HLA-DQB1*, *HLA-DMA*, and *HLA-DRA* (**Supplementary Figure S4**). Similar results were also observed in GSE72094 (**Supplementary Figures S5A–C**) and GSE31210 (**Supplementary Figures S6A–C**). To explore the potential response of patients with LUAD to immunotherapy, we compared the TIDE scores and IPS across different SPRrisk groups in TCGA LUAD dataset. The results indicated that the patients in the low-SPRrisk group had a lower TIDE score and a higher IPS than those in the high-SPRrisk group (**Supplementary Figures S7A–E**). Similarly, the TIDE score distribution plots in two independent datasets (GSE72094 and GSE31210) yielded consistent findings (**Supplementary Figures S7F, G**). Collectively, these results suggested that risk scores may predict the effectiveness of immunotherapy in patients with LUAD.

### Validation of the Nine Secreted Protein-Related Gene Signatures in the Independent Validation Sets

**Figure 6:**
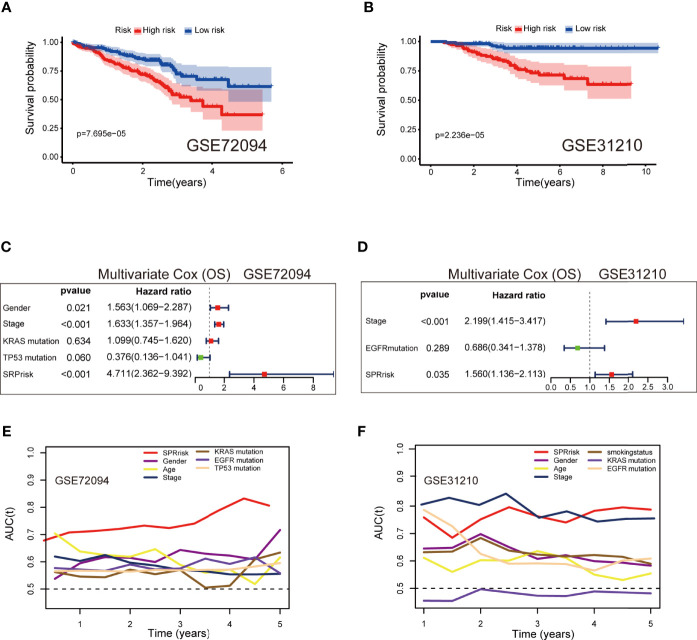
Validation of the 9-gene signature in the independent validation sets. **(A, B)** Kaplan–Meier curves of overall survival time between the high- and low-risk groups using the log-rank test in the GSE72094 and GSE31210 datasets. **(C, D)** Forest plot with hazard ratios from the multivariable Cox proportional hazards regression analysis in the GSE72094 and GSE31210 datasets. **(E, F)** Areas under the curve of time-dependent receiver operating characteristic curves of risk factors in the GSE72094 and GSE31210 datasets.

The baseline characteristics of the patients in the different risk groups in the GSE72094 and GSE31210 datasets are shown in **Supplementary Table S3**. To examine the accuracy of the model constructed based on TCGA testing cohort, we calculated the risk score of each patient in the validation sets according to the formula presented above. Additionally, the patients were divided into high-risk and low-risk groups according to the median risk score. The results were consistent with those of TCGA testing cohort and indicated that the patients in the high-risk group had shorter survival times than those in the low-risk group (**Figures 6A, B**). In the multivariate analyses of the two independent sets, both SPRrisk and stage were independent prognostic risk factors, suggesting the presence of a complementary mechanism (**Figures 6C, D**). Furthermore, in TCGA and independent validation sets, the SPRrisk was consistently an independent prognostic factor after adjustments for the different clinicopathological characteristics (**Supplementary Table S4**). The ROC analysis also showed higher AUCs for SPRrisk, which highlighted the strong prognosis-predicting ability of SPRrisk (**Figures 6E, F**).

### Combination of the Secreted Protein-Related Signature and Clinicopathological Features Improves Survival Prediction

**Figure 7:**
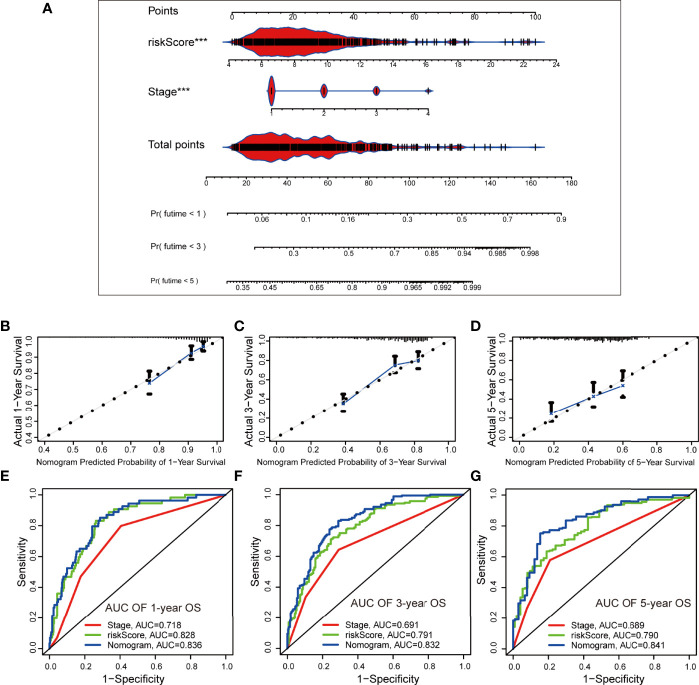
Establishment and evaluation of a nomogram based on independent prognostic factors in The Cancer Genome Atlas cohort. **(A)** The nomogram generated from independent prognostic factors predicts the overall survival (OS) of patients with lung adenocarcinoma. **(B–D)** Calibration plot analyses for the predictive value of prognostic factors. **(E–G)** Comparison of receiver operating characteristic curves of independent prognostic factors in predicting 1-, 3-, and 5-year OS. ***P < 0.001.

A nomogram was established using the SPRrisk and stage as independent prognostic factors in TCGA cohort (**Figure 7A**). Through calculation, the C-index of the prognostic model developed, using TCGA cohort, was 0.784, indicating that the consistency of the model was satisfactory. The calibration curve results demonstrated that the survival status predicted by the prognostic model was in good agreement with the actual survival status (**Figures 7B–D**). In addition, multivariate ROC curves were plotted to compare the AUC of different prognostic factors, and the results showed that the nomogram presented the highest accuracy in predicting 1-, 3-, and 5-year OS (AUC = 0.838, 0.832, and 0.841, respectively) (**Figures 7E–G**). Similarly, we evaluated the ability of the prognostic model to predict the survival status of patients with LUAD in the validation cohorts (GSE72094 and GSE31210). Two nomograms were generated based on the independent prognostic factors to predict the probability of OS (**Supplementary Figures 8A, B**). The C-index values for the two nomograms were 0.753 and 0.768, respectively, and the calibration plots indicated that the predicted survival of the model matched the actual survival (**Supplementary Figures S8C, D**). Additionally, the accuracy of this nomogram in predicting OS was the highest (**Supplementary Figures S8E, F**). Overall, these results suggested that the nomogram has a great potential for predicting the survival and prognosis of patients with LUAD.

A few laboratories have published studies in which they constructed prognostic models to achieve a more accurate evaluation of the prognosis for patients with LUAD—for example, the hypoxia-related risk score (HRrisk) and the tumor microenvironment-related risk score (TMErisk) (45, 46). We compared our model with these existing predictive models in the validation sets (TCGA, GSE72094, and GSE31210) using multivariate Cox regression analysis and time–ROC analysis. In the GSE72094 dataset, the SPRrisk was still confirmed as an independent prognostic factor (**Supplementary Figures S9A, B**). New nomograms were constructed using the SPRrisk and the existing predictive gene signatures (**Supplementary Figures S9C, D**), and the addition of SPRrisk resulted in further improvements in the model’s predictive ability of LUAD prognosis (**Supplementary Figures S9E, F**). In TCGA dataset, only SPRrisk and TMErisk were independent prognostic factors, and the nomogram and the ROC curves revealed that modeling outperformed both separately (**Supplementary Figures S10A–D**). In the GSE31210 dataset, HRrisk and TMErisk were not statistically significant in the multivariate Cox regression analysis (**Supplementary Figures S10E, F**).

### Validation of the Expression Levels of Selected SPRGs

**Figure 8:**
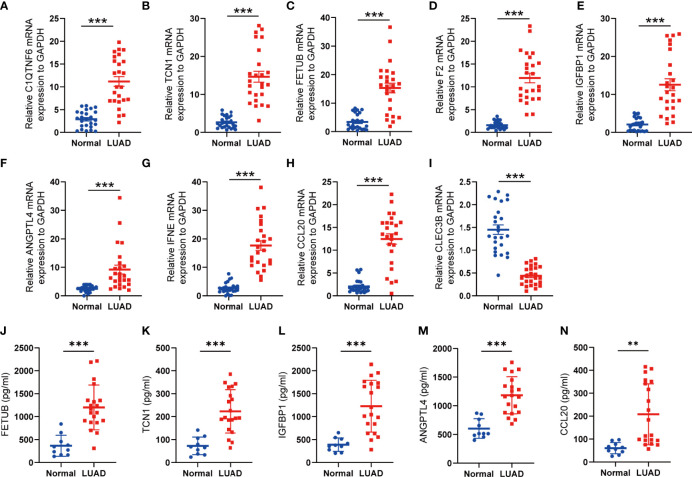
Validation of the expression of SPRGs by qRT-PCR and ELISA. **(A–I)** Relative 9 SPRG mRNA expression between the normal and lung adenocarcinoma. **(J–N)** Plasma levels of the 5 secreted proteins between the normal and lung adenocarcinoma. **P < 0.01, ***P < 0.001 by Student’s *t*-test.

To assess the expression levels of the selected SPRGs within the lung tissue, lung tumor tissues (*n* = 25) and normal lung tissues (*n* = 25) were analyzed by qPCR. Compared to those in the non-tumor lung tissues, the expression levels of *C1QTNF6*, *TCN1*, *F2*, *FETUB*, *IGFBP1*, *ANGPTL4*, *IFNE*, and *CCL20* were upregulated in lung cancer tissues, while the expression level of *CLEC3B* was downregulated (**Figures 8A–I**). To further validate the predictive power of the SPRG signature, ELISA was used to measure the plasma levels of the secreted proteins in our clinical dataset. The baseline characteristics of the patients are shown in **Supplementary Table S5**. Consistent with the qPCR results, the levels of *TCN1*, *FETUB*, *IGFBP1*, *ANGPTL4*, and *CCL20* were significantly elevated in the plasma of patients with LUAD (**Figures 8J–N**). The KM survival analysis showed that stage I patients had an extended survival rate than stage II patients (**Supplementary Figure S11**). Accordingly, differences in plasma levels of secreted proteins between stage I and stage II patients were analyzed using Students *t*-tests, and the results indicated that patients with stage II LUAD had higher plasma protein levels of TCN1, FETUB, IGFBP1, ANGPTL4, and CCL20 (**Table 2**).

**Table 2:** Correlation between the plasma levels of the secreted proteins and the clinical stage.

Parameter	Stage I	Stage II	*P*-value
	(*N* = 13)	(*N* = 7)	
Gender			
Female	6 (46.2%)	5 (71.4%)	0.5402
Male	7 (53.8%)	2 (28.6%)	
Smoking status			
Ever-smoker	7 (53.8%)	1 (14.3%)	0.2131
Never-smoker	6 (46.2%)	6 (85.7%)	
Age (years)			
Mean (SD)	59.1 (7.39)	58.4 (6.29)	0.839
Median (min., max.)	58 (49.0,73.0]	58 (50.0, 69.0)	
ANGPTL4 (pg/ml)			
Mean (SD)	897 (214)	1,270 (391)	0.0451
Median (min., max.)	887 (582, 1,340)	1,160 (798, 1880)	
IGFBP1 (pg/ml)			
Mean (SD)	1,010 (455)	1,680 (421)	0.0052
Median (min., max.)	986 (441, 1,730)	1,770 (847, 2,140)	
CCL20 (pg/ml)			
Mean (SD)	169 (116)	294 (124)	0.0496
Median (min., max.)	95.3 (56.7, 357)	342 (117, 415)	
TCN1 (pg/ml)			
Mean (SD)	183 (81.9)	296 (79.6)	0.0102
Median (min., max.)	185 (48.2, 329)	332 (189, 393)	

## Discussion

Secreted proteins are first synthesized in the cell and then actively secreted to other organelles or the extracellular environment. Secreted proteins include cytokines, growth factors, complement, degradation enzymes, antibodies, peptide hormones, and immunoglobulins, all of which have important physiological functions (47, 48). It is estimated that more than 2,000 proteins in human cells are secreted, and these protein molecules are critical in regulating the physiology and development of organisms. However, the biological functions of these proteins have remained poorly understood (49). Secreted proteins can be classified into classical secreted proteins and non-classical secreted proteins based on whether the N-terminal signal peptides are involved in the protein secretion process or not (50). In our study, we focused on the genes encoding proteins secreted into the plasma; hence, we selected nine SPRGs from the expression profiles of patients with LUAD to construct a prognostic model with good predictive power and specificity. It is worth mentioning that our research is the first to screen differentially expressed genes based on secreted proteins and to build a prognosis model based on these SPRGs for patients with LUAD. Sun *et al*. reported that *CLEC3B*, which encodes tetranectin in humans, was significantly downregulated in patients with lung cancer compared with that in nontumor control groups according to database analysis and patient tissue sample detection (51). Indeed the plasma levels of *CLEC3B* are altered in the blood samples of patients with COVID-19 infection or acute coronary syndrome (52, 53). *C1QTNF6*, encoding C1q/tumor necrosis factor-related protein 6, is a newly identified adiponectin paralog associated with inflammation (54). Zhang et al. found that the inhibition of *C1QTNF6* attenuated cell proliferation, migration, and invasion and promoted apoptosis *in vitro* and *in vivo* in NSCLC (55). TCN1 generates a transcobalamin–cobalamin (vitamin B12) complex and regulates cobalamin homeostasis. It was reported that high levels of TCN1 in human serum are associated with leukemia, hepatocellular carcinoma, and phyllodes of breast tumors (56, 57). As a member of the cysteine protease inhibitor family, FETUB is a glycoprotein. It has been reported that the levels of FETUB are altered in human serum in the process of ischemic stroke or severe COVID-19 (58, 59). IFNE is a type I interferon with unusual patterns of expression and function. Nevertheless, *in vivo* experiments indicated its efficacy in regulating mucosal immune responses and fighting bacterial and viral infections. ANGPTL4 and IGFBP1 are secreted into the plasma and are involved in cell energy metabolism (60–62).

During the development of malignant tumors, tumor cells secrete a variety of proteins, such as cytokines and proteolytic enzymes. The secreted proteins display an altered composition compared to the normal tissue, and their expression levels may change during different tumor stages (63). Consequently, secreted proteins have become the main source of potential tumor markers (64, 65). Since the expression levels of many secretory proteins are altered in tumors and these altered levels can be easily detected in body fluids, secretory proteins have good diagnostic and prognostic values. Some well-known secreted tumor markers include AFP, cell surface-associated protein (MUC1 or CA15-3), gastrin-releasing peptide, and prostate-specific antigen (or KLK3) (66–69). In our study, we conducted ELISA to examine the levels of several secreted proteins in the peripheral blood of patients with LUAD. We found that the levels of candidate secreted proteins were positively correlated with the clinical stage of the patients with LUAD and agreed with the model results. Our findings highlighted the potential for the selected secreted proteins to serve as a prognostic marker for human LUAD. Detecting secreted protein levels in body fluids is economical, quantitative, and minimally invasive compared with RNA-seq, and more people may benefit from our study.

In addition, some secreted proteins play a significant role in regulating the immune microenvironment, which makes them potential targets for tumor therapy. Gelsolin (GSN) was reportedly secreted by cancer cells, which suppressed the killing activity of CD8^+^ T cells against tumor cells. Moreover, lower levels of intratumoral GSN transcripts are associated with signatures of anti-cancer immunity and increased patient survival (70). Tumor cells also secrete proteins, such as IL-10 and TGF-β, to remodel the immune microenvironment and promote tumor progression (71). Chemotherapy or radiotherapy can also induce senescence in tumor cells by modifying their secretome to a “senescence-associated secretory phenotype”, which also affects the immune response (72). In our study, we grouped the patients with LUAD into high- and low-risk groups based on the risk score. We found increased CD8^+^ T cell and M1 macrophage cell infiltration in the low-risk group, while the high-risk group showed a higher M2 macrophage cell infiltration. CD8^+^ T cells are the primary mediators of anticancer immunity, and the modulation of the CD8^+^ T cell response has been a central focus of immunotherapy to treat cancer (73). Macrophages within the tumor stroma are tumor-associated macrophages and can be categorized as either classically activated M1 or alternatively activated M2 macrophages (74). M1 macrophages are considered anti-tumorous as they kill tumor cells by producing pro-inflammatory cytokines, such as IL-1β and IL-12. In contrast, M2 macrophages are considered pro-tumorous since they stimulate the secretion of anti-inflammatory cytokines, such as IL-10, IL-13, and TGF-β (75). By estimating multiple published transcriptomic biomarkers based on pre-treatment tumor expression profiles, TIDE scores can predict patient response to immunotherapies (39). Our current findings also revealed that the low-risk group achieved a higher TIDE score than the high-risk group. The IPS was a superior predictor of response to anti-CTLA-4 and anti-PD-1 antibodies (38). Interestingly, significant differences in different IPS between the high- and low-risk groups were indicated. Thus, our risk model based on SPRGs could be used to predict the immunotherapy response rates and present the most appropriate therapeutic options for patients with LUAD—for example, for the low-risk group with increased infiltration of CD8^+^ T cells and M1 macrophages, the immune checkpoint inhibitors may turn out to be effective treatments.

It is incontrovertible that this study has some limitations. First, various deficiencies in clinical information led to the incomplete validation of the partial results in TCGA LUAD training set and GEO verification set. Second, the number of available samples and clinical specimens was insufficient for conducting ELISA and comprehensive molecular studies, respectively. More tissue samples will be needed in further studies for validation. Third, the SPRGs were identified and validated using retrospective data from public databases. However, validation using a larger number of cases in a prospective cohort study is needed in the future. Finally, the molecular mechanism has not been characterized, and additional experiments are needed to explore the mechanistic roles of SPRGs in tumor progression.

In summary, our study enriches the current knowledge on the use of SPRGs for the prognostic prediction of LUAD. The prognostic SPRG model constructed in our study exhibited a robust capacity in predicting the survival outcomes of patients with LUAD and was correlated with the immune landscape of the LUAD microenvironment. We hope that these findings will offer useful insights for future studies and clinical practices.

## Data Availability Statement

The datasets presented in this study can be found in online repositories. The names of the repository/repositories and accession number(s) can be found in the article/**Supplementary Material**.

## Ethics Statement

The studies involving human participants were reviewed and approved by The Medical Ethics Committee of Union Hospital, Tongji Medical College, Huazhong University of Science and Technology. The patients/participants provided their written informed consent to participate in this study.

## Author Contributions

SC and JZ conceived and designed the study. QL and LX collected and analyzed the related data. XF, QN, and LZ edited and wrote the draft. WM and HY revised the manuscript. All authors contributed to the article and approved the submitted version.

## Funding

This work was supported by the National Natural Science Foundation of China (numbers 81973991 and 91643101 to WLM and numbers 82070066 and 81873401 to HY).

## Conflict of Interest

The authors declare that the research was conducted in the absence of any commercial or financial relationships that could be construed as a potential conflict of interest.

## Publisher’s Note

All claims expressed in this article are solely those of the authors and do not necessarily represent those of their affiliated organizations, or those of the publisher, the editors and the reviewers. Any product that may be evaluated in this article, or claim that may be made by its manufacturer, is not guaranteed or endorsed by the publisher.

## Supplementary Material

Supplementary Figure 1 Forest plot depicting the result of the univariate Cox regression analysis.

Supplementary Figure 2 Survival analysis of overall survival in patients with lung adenocarcinoma. **(A–I)** Kaplan–Meier survival analysis of 9 selected secreted protein-related genes (SPRGs), respectively. The patients were stratified into high- and low-expression subgroups using the medians of the SPRGs.

Supplementary Figure 3 Prognostic value of SPRS in predicting disease-free interval (DFI), disease-specific survival (DSS), and progression-free interval (PFI). **(A–C)** Kaplan–Meier survival analysis of SPRS for FI, DSS, and PFI in TCGA cohort.

Supplementary Figure 4 Expression of HLA family genes. Box plots depicting the HLA family genes’ expression of high- and low-risk groups in TCGA cohort. The red box plots indicate the SPRrisk-high group, while the blue box plots indicate the SPRrisk-low group.

Supplementary Figure 5 Landscape of immune cell infiltrations in GSE72094. **(A)** Immune cell infiltration levels of 22 immune cell types between the SPRrisk-high and SPRrisk-low groups for patients with lung adenocarcinoma. **(B)** Analyses for the expression of immune checkpoint genes in the SPRrisk-high and SPRrisk-low groups. **(C)** Analyses for the expression of human leukocyte antigen family genes in the SPRrisk-high and SPRrisk-low groups.

Supplementary Figure 6 Landscape of immune cell infiltrations in GSE31210. **(A)** Immune cell infiltration levels of 22 immune cell types between these risk-high and SPRrisk-low groups for patients with lung adenocarcinoma. **(B)** Analyses for the expression of immune checkpoint genes in the SPRrisk-high and SPRrisk-low groups. **(C)** Analyses for the expression of human leukocyte antigen family genes in the SPRrisk-high and SPRrisk-low groups.

Supplementary Figure 7 Distribution of the tumor immune dysfunction and exclusion (TIDE) scores and immunophenoscore (IPS) scores across different SPRrisk groups. **(A–D)** IPS score, IPS–CTLA4 blocker score, IPS–CTLA4 blocker score, and IPS–CTLA4 and PD1/PDL1/PDL2 blocker score distribution plots in The Cancer Genome Atlas (TCGA) training dataset. **(E)** TIDE score distribution plot in TCGA lung adenocarcinoma dataset. **(F)** TIDE score distribution plot in GSE72094 dataset. **(G)** TIDE score distribution plot in GSE31210 dataset.

Supplementary Figure 8 Nomogram based on independent prognostic factors for overall survival (OS) of patients with lung adenocarcinoma (LUAD) in the independent validation sets. **(A, B)** The nomogram generated from independent prognostic factors predicts the OS of patients with LUAD in GSE72094 and GSE31210. **(C, D)** Calibration plot analyses for the predictive value of prognostic factors in the GSE72094 and GSE31210 datasets. **(E, F)** Comparison of receiver operating characteristic curves of independent prognostic factors in predicting the OS in the GSE72094 and GSE31210 datasets.

Supplementary Figure 9 Assessment of the predictive ability of SPRrisk with the existing predictive models in GSE72094. **(A)** Multivariable Cox proportional hazards regression analysis of SPRrisk and TMErisk in the GSE72094 dataset. **(B)** Multivariable Cox proportional hazards regression analysis of SPRrisk and HRrisk in the GSE72094 dataset. **(C)** The nomogram generated from SPRrisk and TMErisk predicts the overall survival (OS) of patients in GSE72094. **(D)** The nomogram generated from SPRrisk and HRrisk predicts the OS of patients in GSE72094. **(E)** The areas under the curve (AUCs) of time-dependent receiver operating characteristic (ROC) curves verified the prognostic performance of SPRrisk and TMErisk in GSE72094. **(F)** The AUCs of time-dependent ROC curves verified the prognostic performance of the SPRrisk and HRrisk in GSE72094.

Supplementary Figure 10 Assessment of the predictive ability of SPRrisk with the existing predictive models in The Cancer Genome Atlas (TCGA) and GSE31210 datasets. **(A)** Multivariable Cox proportional hazards regression analysis of SPRrisk and HRrisk in TCGA dataset. **(B)** Multivariable Cox proportional hazards regression analysis of SPRrisk and TMErisk in TCGA dataset. **(C)** The nomogram generated from SPRrisk and TMErisk predicts the overall survival of patients in TCGA dataset. **(D)** The areas under the curve of time-dependent receiver operating characteristic curves verified the prognostic performance of the SPRrisk and TMErisk in TCGA dataset. **(E)** Multivariable Cox proportional hazards regression analysis of SPRrisk and HRrisk in the GSE31210 dataset. **(F)** Multivariable Cox proportional hazards regression analysis of SPRrisk and TMErisk in the GSE31210 dataset.

Supplementary Figure 11 Survival analysis of overall survival (OS) in patients with lung adenocarcinoma in our dataset. Kaplan–Meier survival analysis of clinical stage for OS in our dataset.
