# Supplementary figures and images for: A Novel Secreted Protein-Related Gene Signature Predicts Overall Survival and Is Associated With Tumor Immunity in Patients With Lung Adenocarcinoma
Source: Front Oncol. 2022 Jun 3;12:870328. doi: 10.3389/fonc.2022.870328 (PMC9204015; doi:10.3389/fonc.2022.870328)

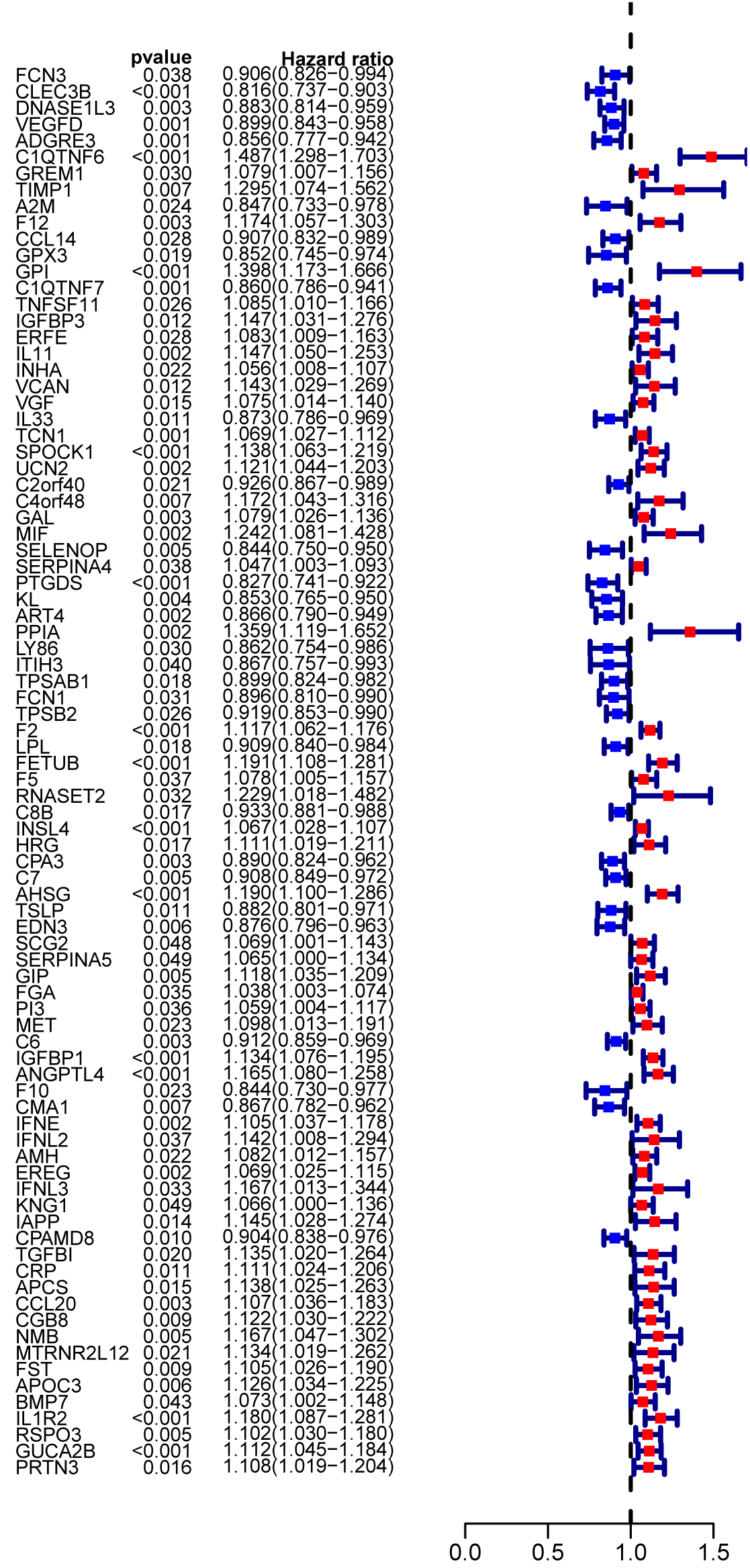

Supplement: Supplementary Figure 1 — Forest plot depicting the result of the univariate Cox regression analysis. [file Image_1.tif]

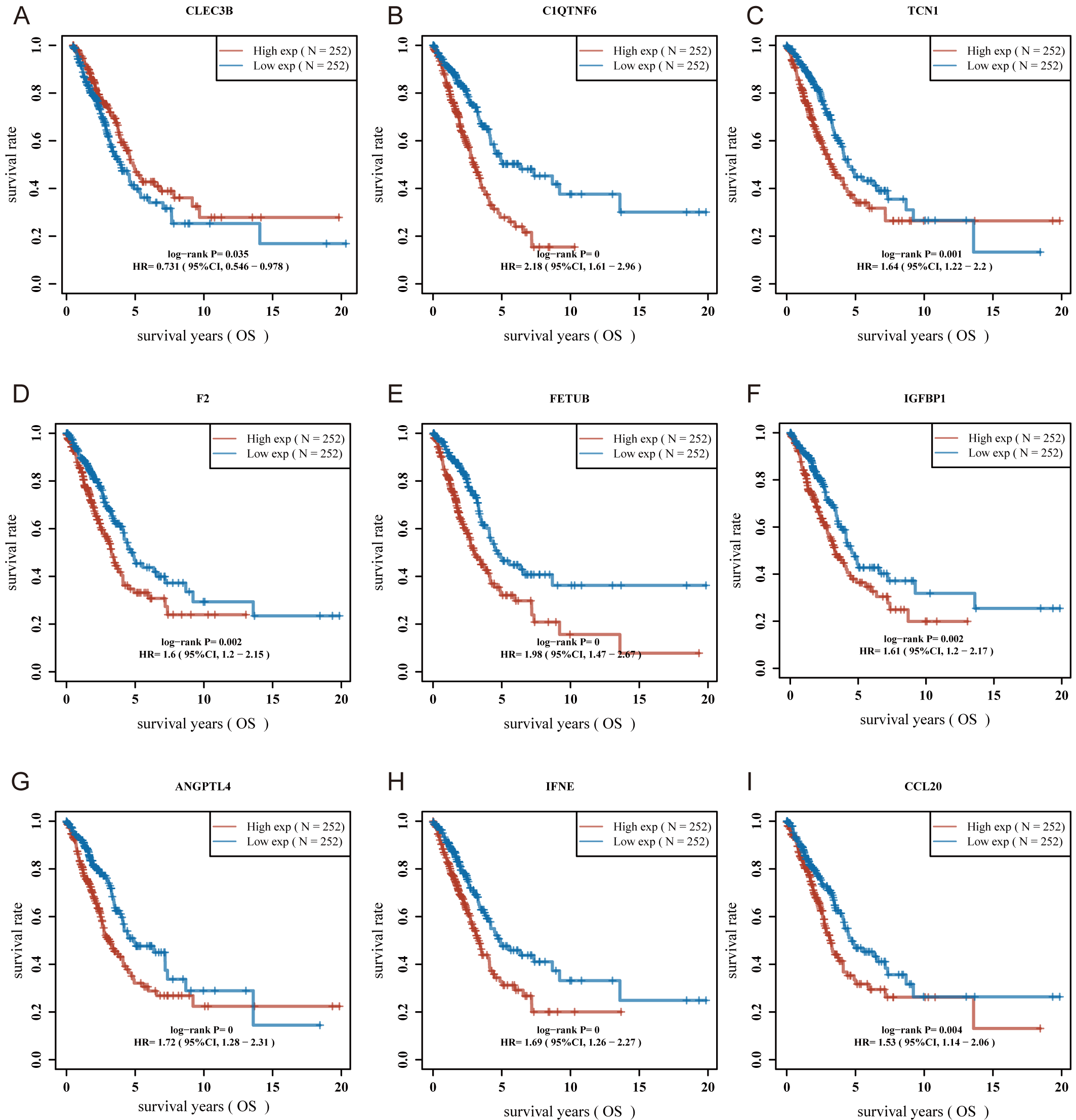

Supplement: Supplementary Figure 2 — Survival analysis of overall survival in patients with lung adenocarcinoma. (A–I) Kaplan–Meier survival analysis of 9 selected secreted protein-related genes (SPRGs), respectively. The patients were stratified into high- and low-expression subgroups using the medians of the SPRGs. [file Image_2.tif]

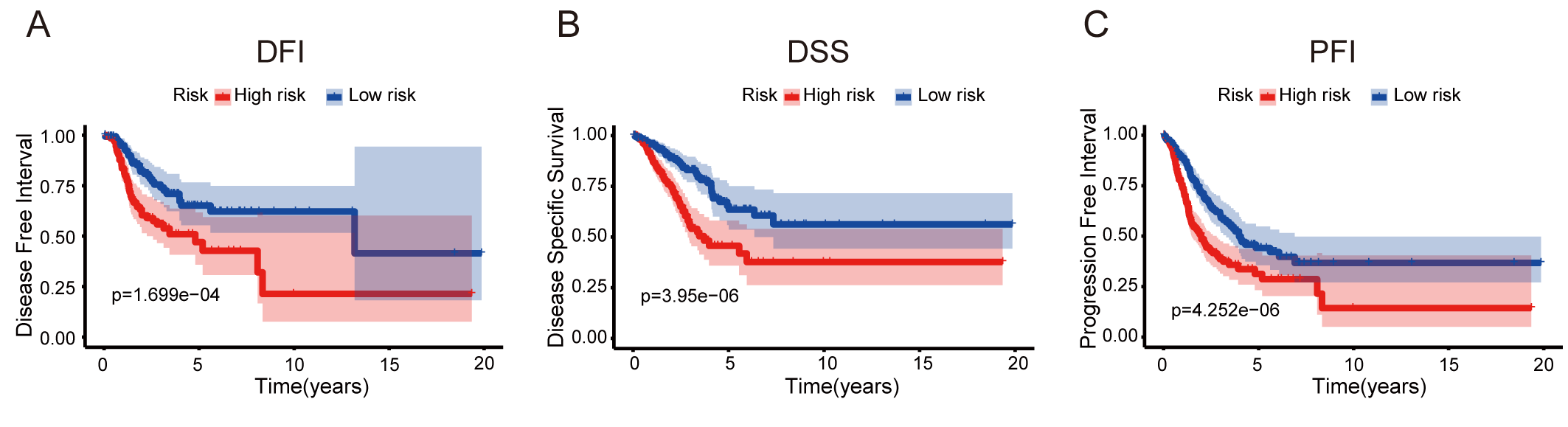

Supplement: Supplementary Figure 3 — Prognostic value of SPRS in predicting disease-free interval (DFI), disease-specific survival (DSS), and progression-free interval (PFI). (A–C) Kaplan–Meier survival analysis of SPRS for FI, DSS, and PFI in TCGA cohort. [file Image_3.tif]

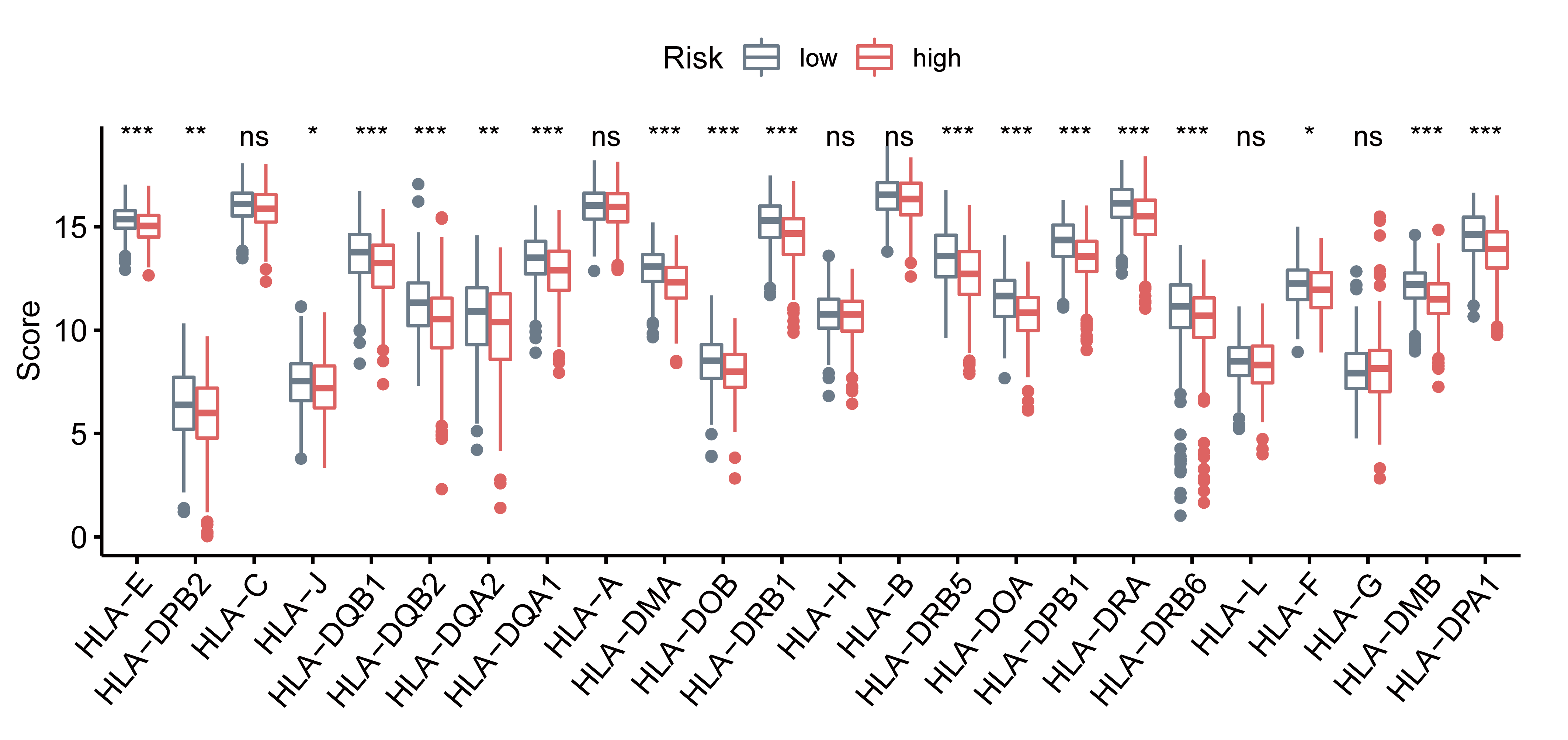

Supplement: Supplementary Figure 4 — Expression of HLA family genes. Box plots depicting the HLA family genes’ expression of high- and low-risk groups in TCGA cohort. The red box plots indicate the SPRrisk-high group, while the blue box plots indicate the SPRrisk-low group. [file Image_4.tif]

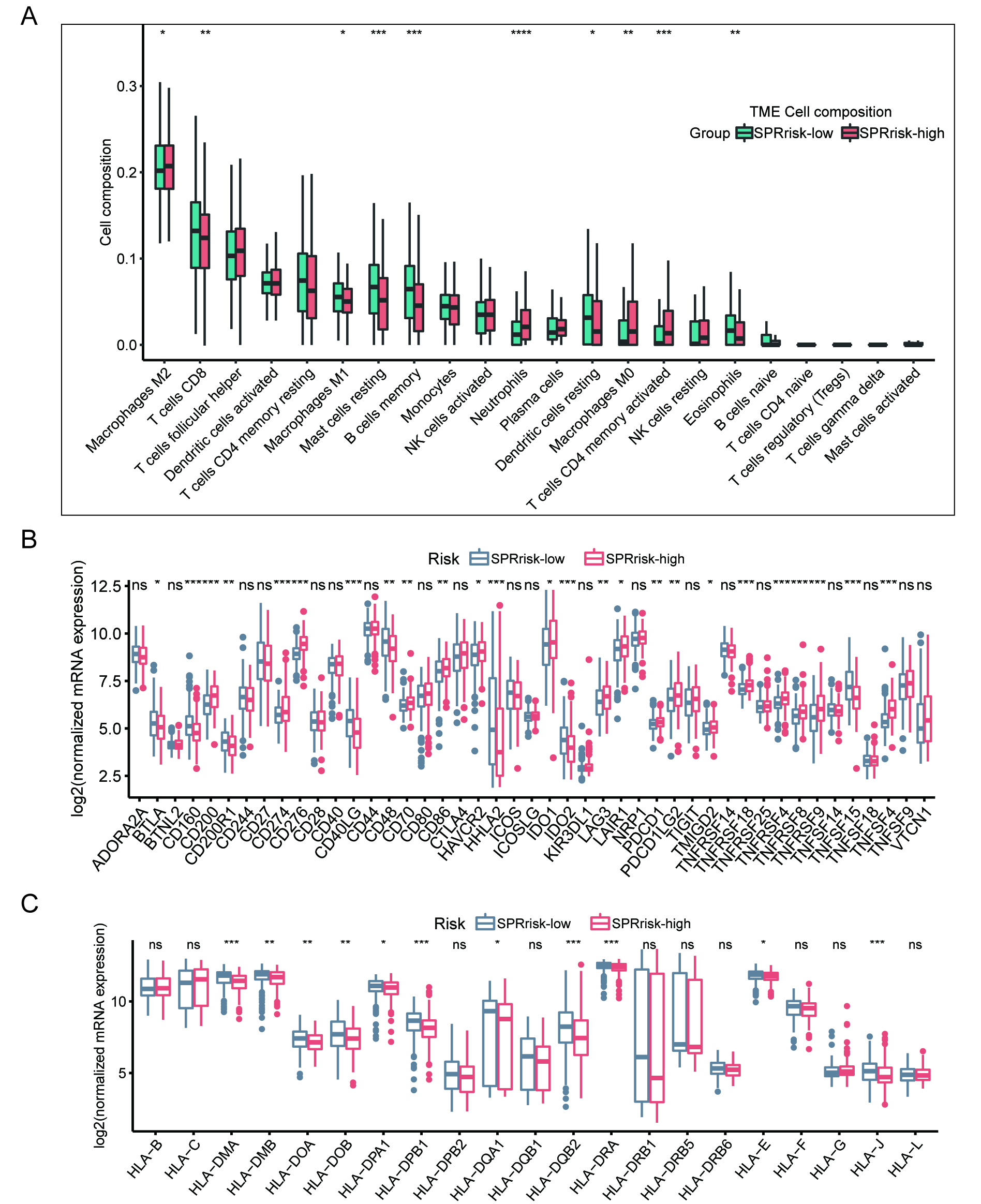

Supplement: Supplementary Figure 5 — Landscape of immune cell infiltrations in GSE72094. (A) Immune cell infiltration levels of 22 immune cell types between the SPRrisk-high and SPRrisk-low groups for patients with lung adenocarcinoma. (B) Analyses for the expression of immune checkpoint genes in the SPRrisk-high and SPRrisk-low groups. (C) Analyses for the expression of human leukocyte antigen family genes in the SPRrisk-high and SPRrisk-low groups. [file Image_5.tif]

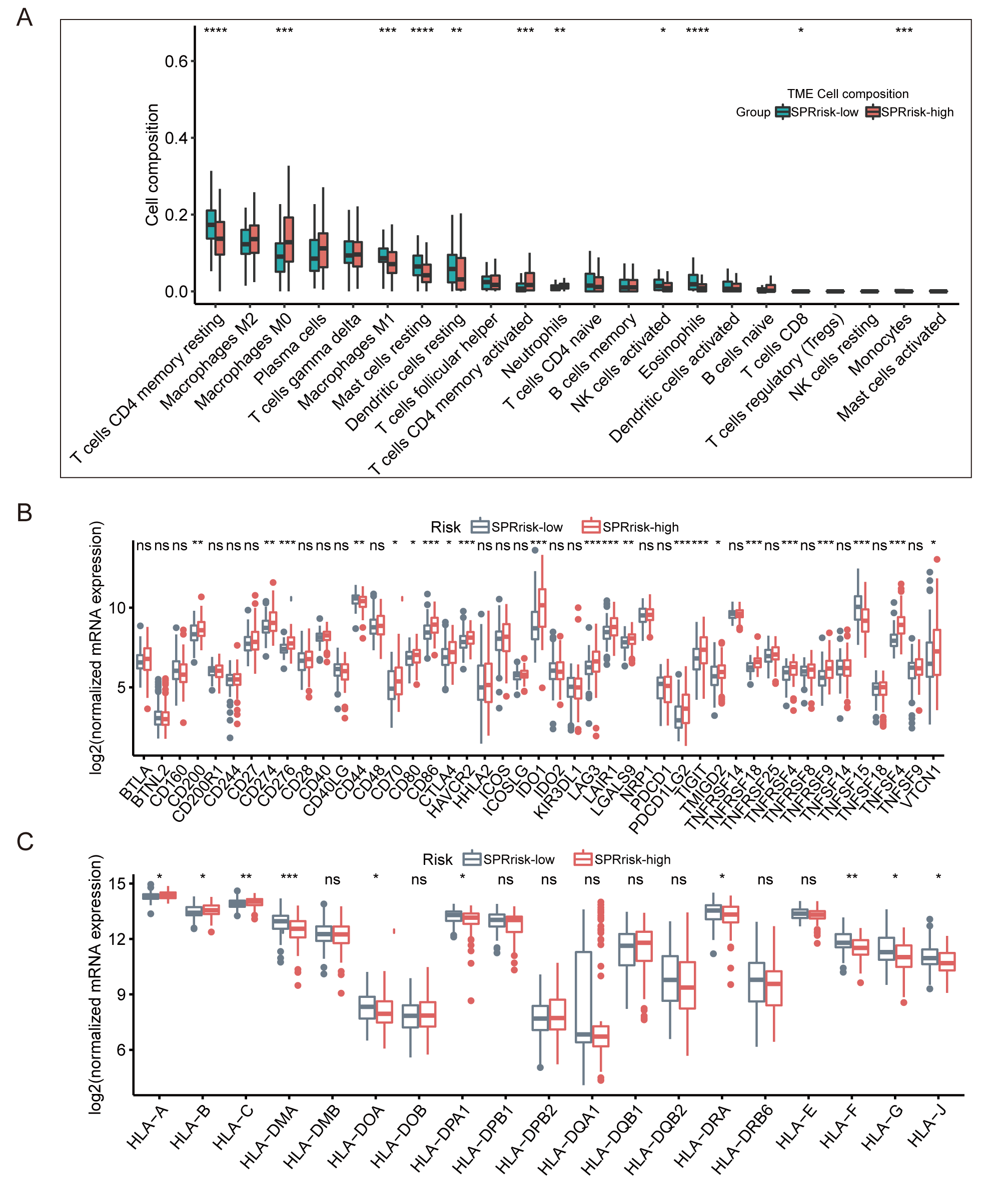

Supplement: Supplementary Figure 6 — Landscape of immune cell infiltrations in GSE31210. (A) Immune cell infiltration levels of 22 immune cell types between these risk-high and SPRrisk-low groups for patients with lung adenocarcinoma. (B) Analyses for the expression of immune checkpoint genes in the SPRrisk-high and SPRrisk-low groups. (C) Analyses for the expression of human leukocyte antigen family genes in the SPRrisk-high and SPRrisk-low groups. [file Image_6.tif]

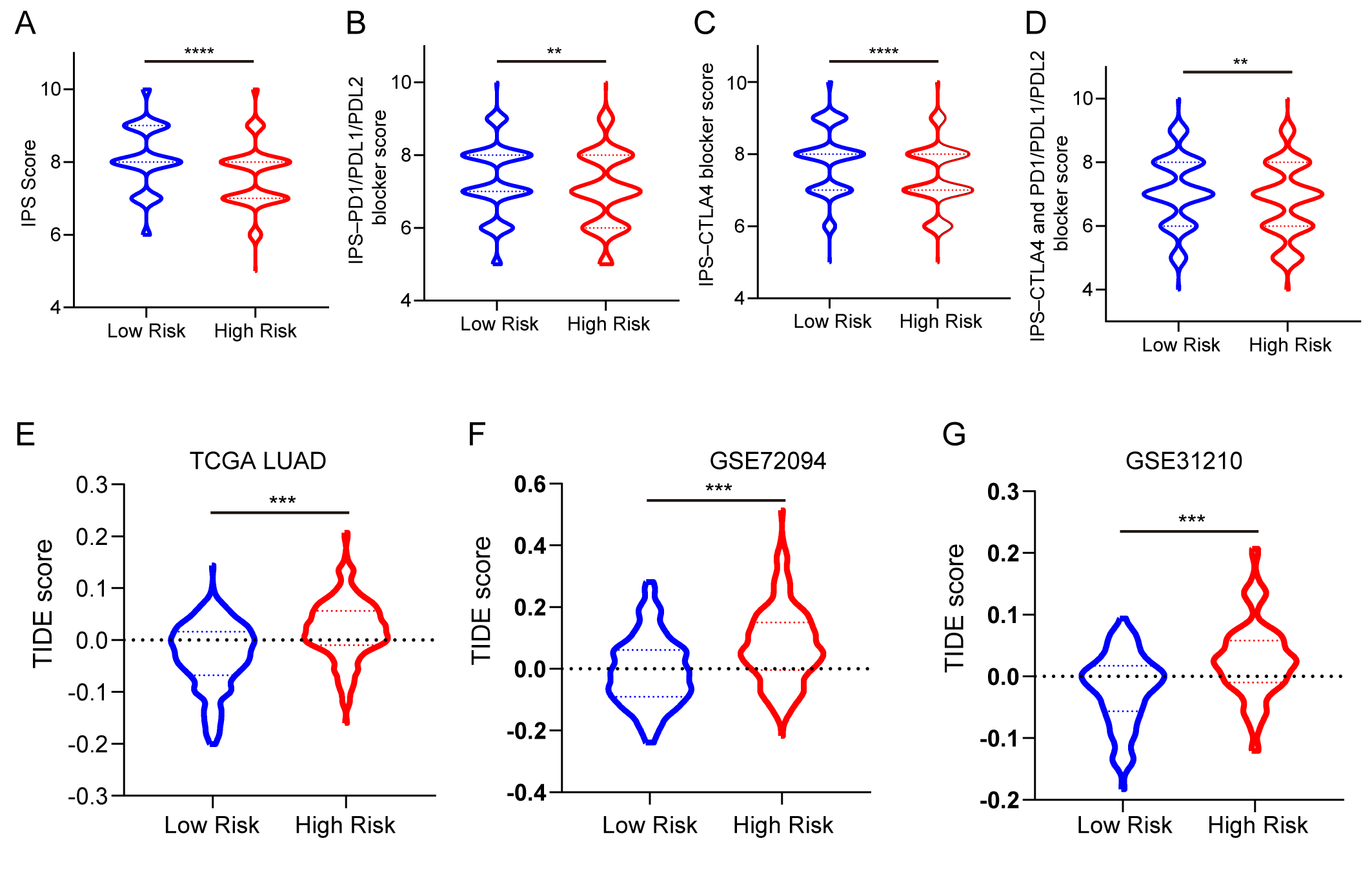

Supplement: Supplementary Figure 7 — Distribution of the tumor immune dysfunction and exclusion (TIDE) scores and immunophenoscore (IPS) scores across different SPRrisk groups. (A–D) IPS score, IPS–CTLA4 blocker score, IPS–CTLA4 blocker score, and IPS–CTLA4 and PD1/PDL1/PDL2 blocker score distribution plots in The Cancer Genome Atlas (TCGA) training dataset. (E) TIDE score distribution plot in TCGA lung adenocarcinoma dataset. (F) TIDE score distribution plot in GSE72094 dataset. (G) TIDE score distribution plot in GSE31210 dataset. [file Image_7.tif]

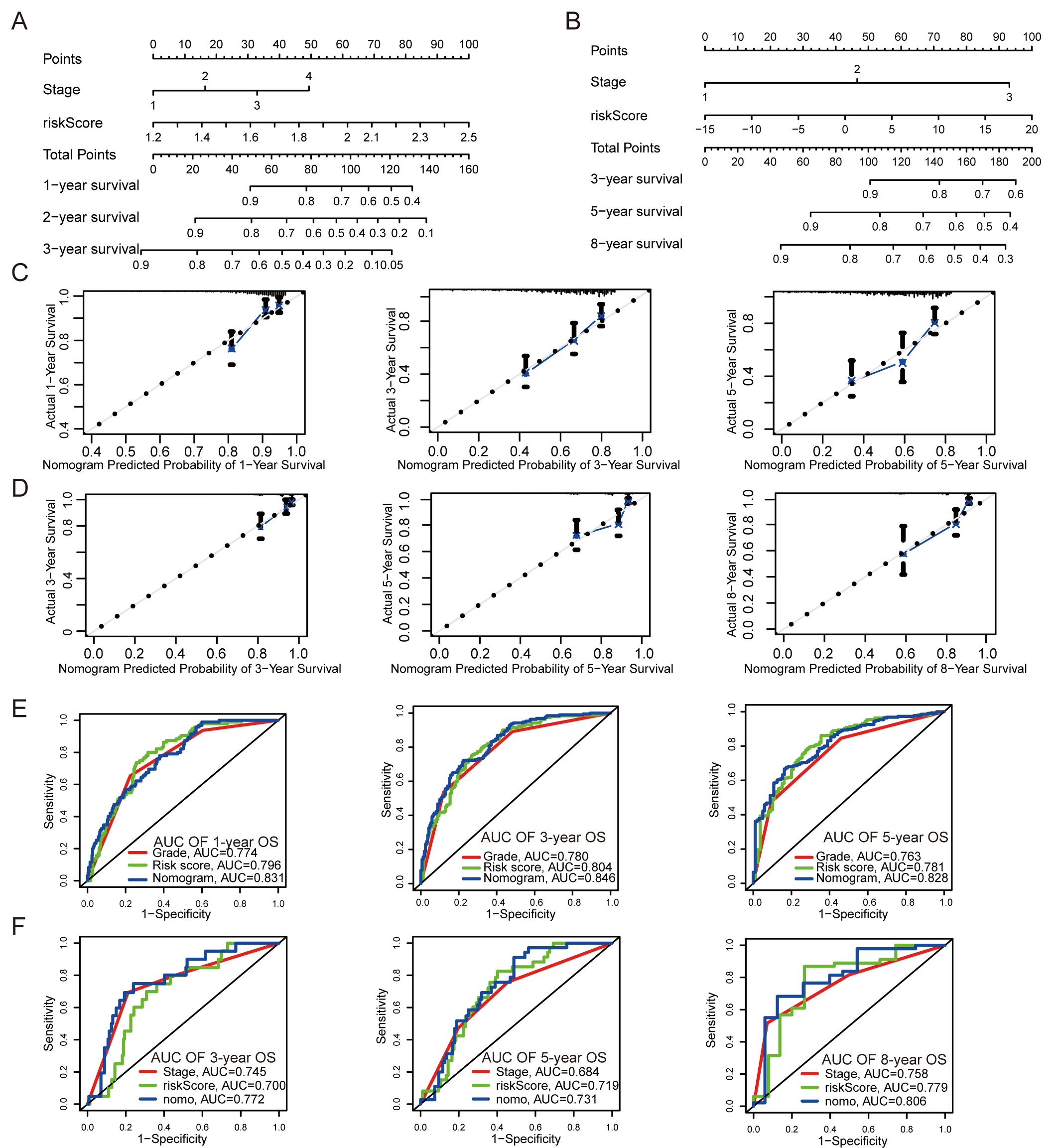

Supplement: Supplementary Figure 8 — Nomogram based on independent prognostic factors for overall survival (OS) of patients with lung adenocarcinoma (LUAD) in the independent validation sets. (A, B) The nomogram generated from independent prognostic factors predicts the OS of patients with LUAD in GSE72094 and GSE31210. (C, D) Calibration plot analyses for the predictive value of prognostic factors in the GSE72094 and GSE31210 datasets. (E, F) Comparison of receiver operating characteristic curves of independent prognostic factors in predicting the OS in the GSE72094 and GSE31210 datasets. [file Image_8.tif]

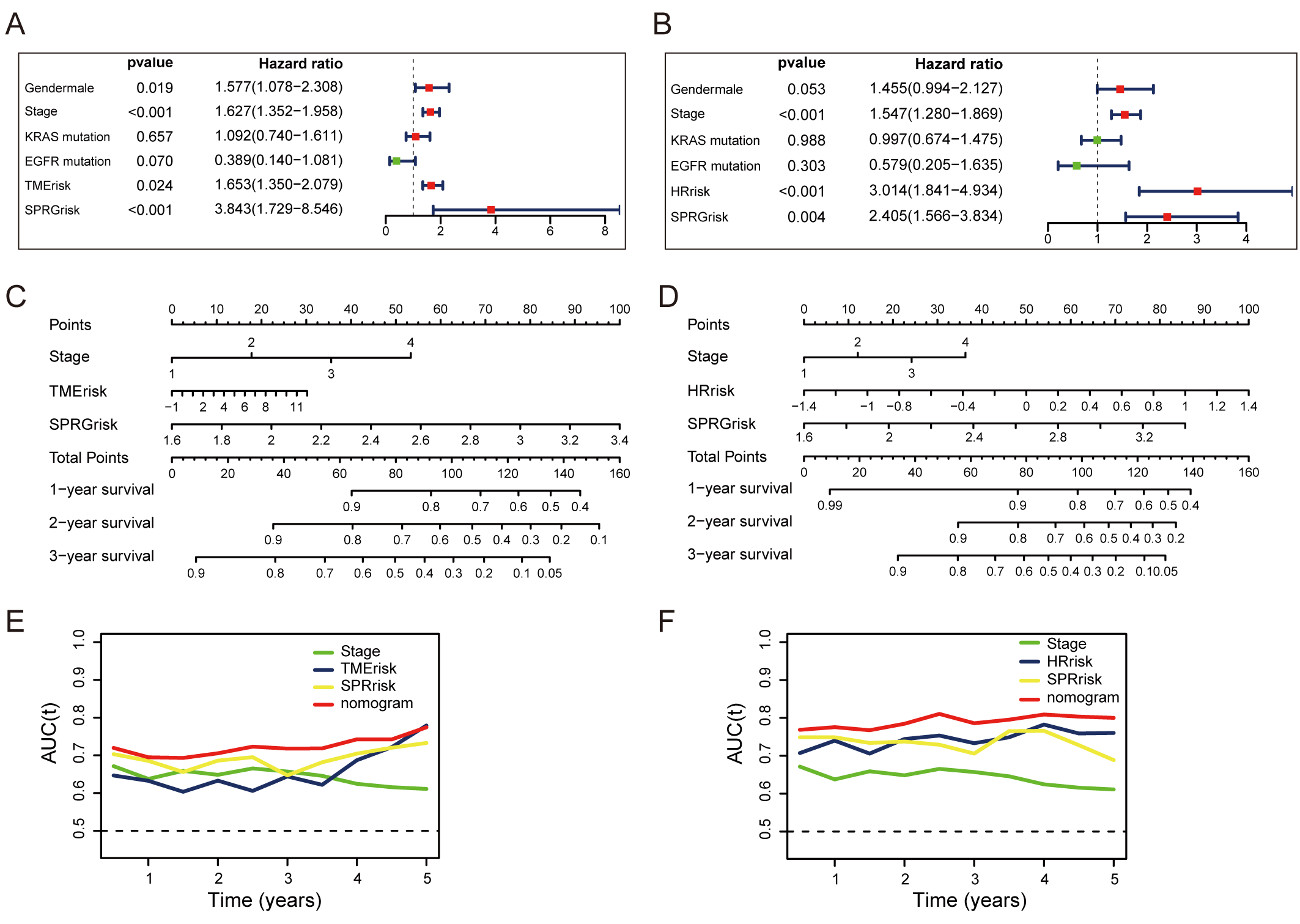

Supplement: Supplementary Figure 9 — Assessment of the predictive ability of SPRrisk with the existing predictive models in GSE72094. (A) Multivariable Cox proportional hazards regression analysis of SPRrisk and TMErisk in the GSE72094 dataset. (B) Multivariable Cox proportional hazards regression analysis of SPRrisk and HRrisk in the GSE72094 dataset. (C) The nomogram generated from SPRrisk and TMErisk predicts the overall survival (OS) of patients in GSE72094. (D) The nomogram generated from SPRrisk and HRrisk predicts the OS of patients in GSE72094. (E) The areas under the curve (AUCs) of time-dependent receiver operating characteristic (ROC) curves verified the prognostic performance of SPRrisk and TMErisk in GSE72094. (F) The AUCs of time-dependent ROC curves verified the prognostic performance of the SPRrisk and HRrisk in GSE72094. [file Image_9.tif]

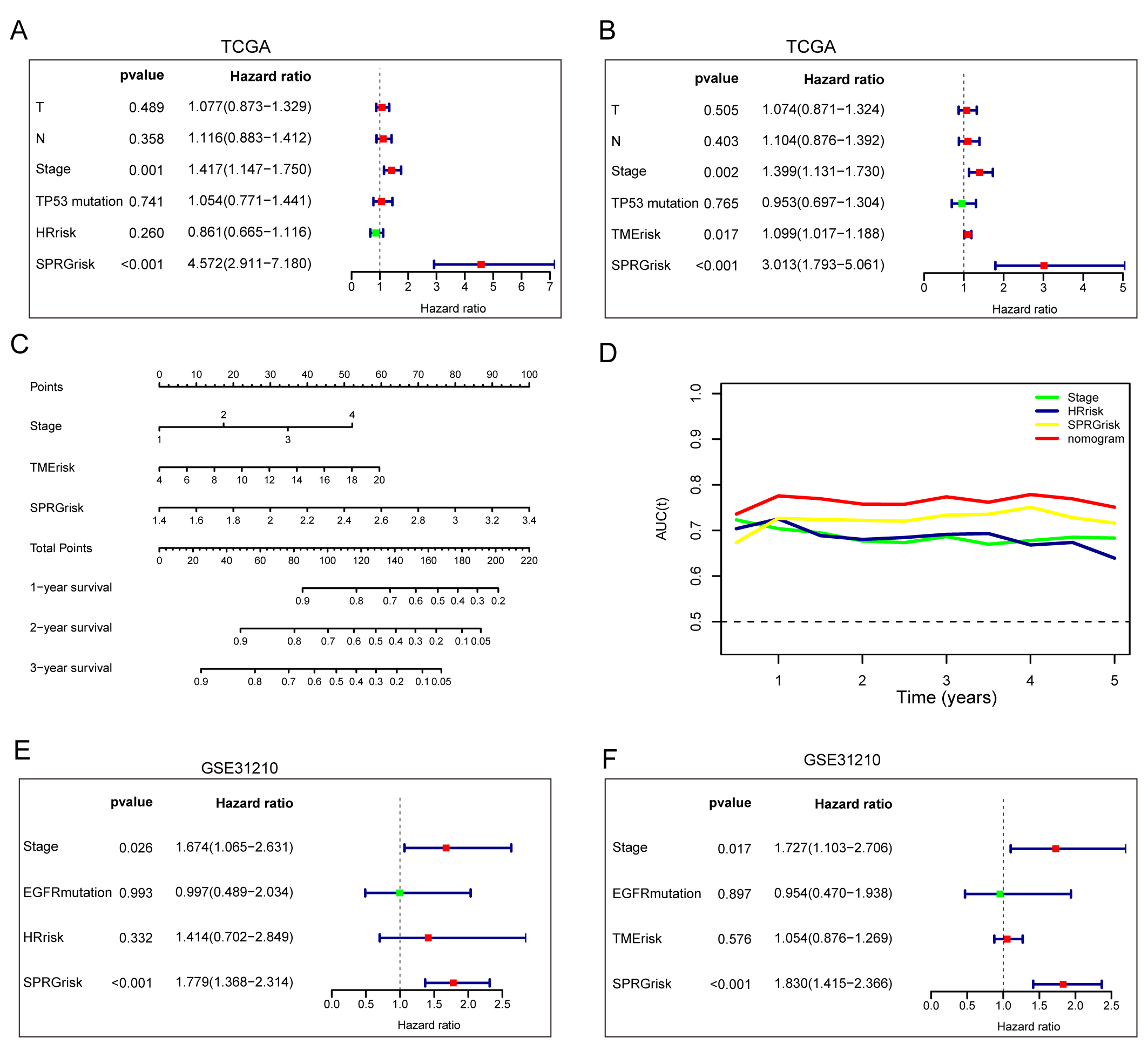

Supplement: Supplementary Figure 10 — Assessment of the predictive ability of SPRrisk with the existing predictive models in The Cancer Genome Atlas (TCGA) and GSE31210 datasets. (A) Multivariable Cox proportional hazards regression analysis of SPRrisk and HRrisk in TCGA dataset. (B) Multivariable Cox proportional hazards regression analysis of SPRrisk and TMErisk in TCGA dataset. (C) The nomogram generated from SPRrisk and TMErisk predicts the overall survival of patients in TCGA dataset. (D) The areas under the curve of time-dependent receiver operating characteristic curves verified the prognostic performance of the SPRrisk and TMErisk in TCGA dataset. (E) Multivariable Cox proportional hazards regression analysis of SPRrisk and HRrisk in the GSE31210 dataset. (F) Multivariable Cox proportional hazards regression analysis of SPRrisk and TMErisk in the GSE31210 dataset. [file Image_10.tif]

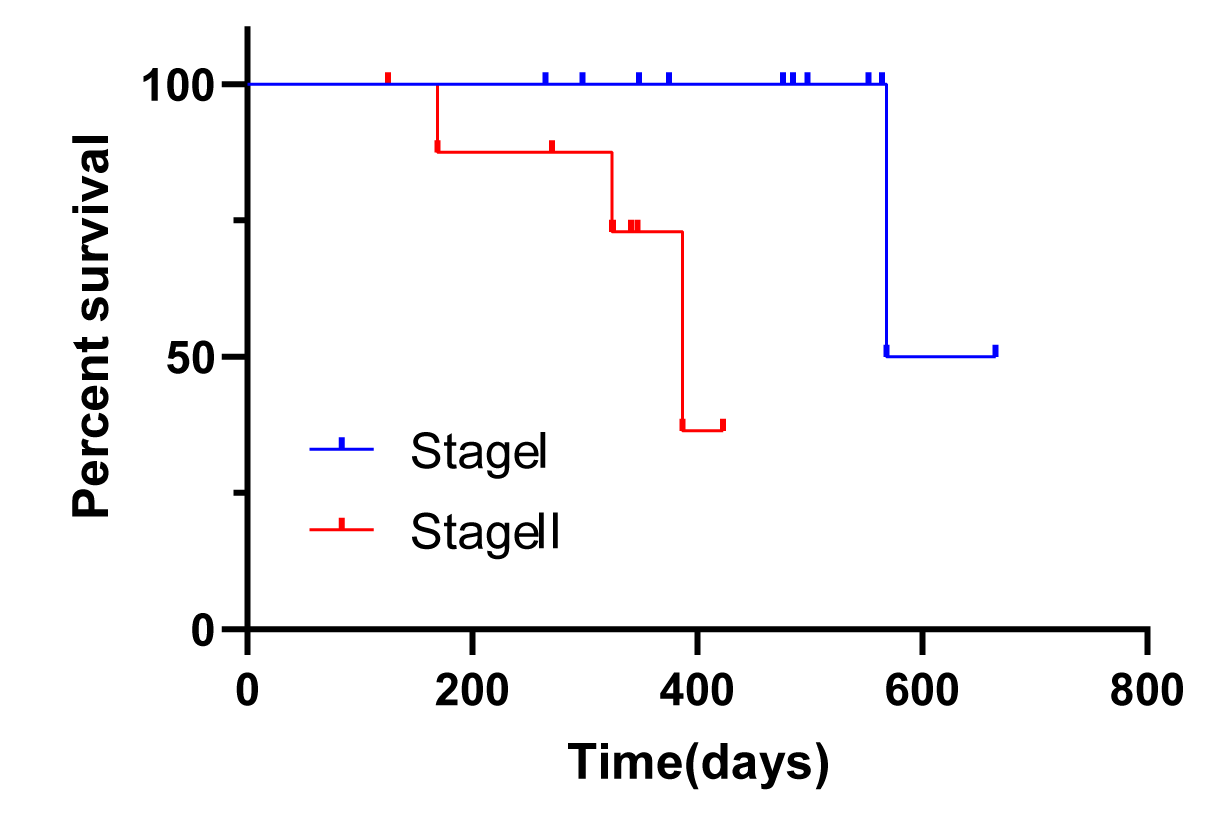

Supplement: Supplementary Figure 11 — Survival analysis of overall survival (OS) in patients with lung adenocarcinoma in our dataset. Kaplan–Meier survival analysis of clinical stage for OS in our dataset. [file Image_11.tif]
